# Improving Motor Activity Assessment in Depression: Which Sensor Placement, Analytic Strategy and Diurnal Time Frame Are Most Powerful in Distinguishing Patients from Controls and Monitoring Treatment Effects

**DOI:** 10.1371/journal.pone.0124231

**Published:** 2015-04-17

**Authors:** Markus Reichert, Alexander Lutz, Michael Deuschle, Maria Gilles, Holger Hill, Matthias F. Limberger, Ulrich W. Ebner-Priemer

**Affiliations:** 1 Department of Sports and Sports Science, Karlsruhe Institute of Technology (KIT), Baden-Wuerttemberg, Germany; 2 Department of Psychiatry and Psychotherapy, Central Institute of Mental Health, Mannheim, Baden-Wuerttemberg, Germany; 3 Department of Psychiatry and Psychotherapy, Central Institute of Mental Health, University of Heidelberg, Medical Faculty Mannheim, Mannheim, Baden-Wuerttemberg, Germany; 4 Department of Psychosomatic Medicine and Psychotherapy, Central Institute of Mental Health, Mannheim, Baden-Wuerttemberg, Germany; University Medical Center Goettingen, GERMANY

## Abstract

**Background:**

Abnormalities in motor activity represent a central feature in major depressive disorder. However, measurement issues are poorly understood, limiting the use of objective measurement of motor activity for diagnostics and treatment monitoring.

**Methods:**

To improve measurement issues, especially sensor placement, analytic strategies and diurnal effects, we assessed motor activity in depressed patients at the beginning (MD; n=27) and after anti-depressive treatment (MD-post; n=18) as well as in healthy controls (HC; n=16) using wrist- and chest-worn accelerometers. We performed multiple analyses regarding sensor placements, extracted features, diurnal variation, motion patterns and posture to clarify which parameters are most powerful in distinguishing patients from controls and monitoring treatment effects.

**Results:**

Whereas most feature-placement combinations revealed significant differences between groups, acceleration (wrist) distinguished MD from HC (d=1.39) best. Frequency (vertical axis chest) additionally differentiated groups in a logistic regression model (R2=0.54). Accordingly, both amplitude (d=1.16) and frequency (d=1.04) showed alterations, indicating reduced and decelerated motor activity. Differences between MD and HC in gestures (d=0.97) and walking (d=1.53) were found by data analysis from the wrist sensor. Comparison of motor activity at the beginning and after MD-treatment largely confirms our findings.

**Limitations:**

Sample size was small, but sufficient for the given effect sizes. Comparison of depressed in-patients with non-hospitalized controls might have limited motor activity differences between groups.

**Conclusions:**

Measurement of wrist-acceleration can be recommended as a basic technique to capture motor activity in depressed patients as it records whole body movement and gestures. Detailed analyses showed differences in amplitude and frequency denoting that depressed patients walked less and slower.

## Introduction

Abnormalities in motor activity do not only reflect the classification criterion of psychomotor retardation in major depressive disorder (MDD), but are also related to other criteria like diminished interest in daily life activities [1,2]. Accordingly, reduced motor activity has been shown in patients with current MDD [3–6] and treatment response was related to increased motor activity [5,7–10].

Fortunately, the use of objective measurement of motor activity has increased in recent years. Technological progress has led to miniaturised accelerative devices which can assess motor activity with high reliability and validity [11,12]. In contrast, meta-analyses revealed only marginal validity of recall questionnaires [13]. In line with this, recent long-term tele-health approaches already use objectively monitored motor activity as a proxy for depressive symptomatology [14–16].

In a recent quantitative review, Burton et al. [17] summarised existing studies using actigraphy to objectively measure motor activity in depressed patients. The review included 19 papers with 16 studies with a total of 412 mostly female patients of various ages. 11 papers dealt with case-control studies and 10 papers referred to longitudinal studies. Participants were diagnosed with MDD (8 papers), depression in association with bipolar disorder (8 papers) or Seasonal Affective Disorder (3 papers). Total assessment period ranged between 2 and 30 days with daytime (15 papers) as well as sleep actigraphy data (14 papers) [17]. Overall, Burton et al. [17] reported less daytime activity in patients with depression compared to healthy controls. Furthermore, treatment studies in depression showed moderate increases in daytime activity over the course of treatment as well as a reduction of night-time activity.

Most importantly, Burton et al. [17] highlighted three methodological limitations of existing studies, which need to be addressed to increase the usefulness of assessing motor activity. First, there is no research and no recommendations regarding the placement of the sensor. Nearly all studies covered in the review used actigraphy devices which were attached to patients’ wrist. Only one study used accelerometers attached to the chest. This is surprising as attaching the device to the centre of mass, the chest or the waist, is the recommendation in the area of motor activity and exercise [18]. Currently, the effect of sensor placement is largely unknown. Thus, having information about different actigraphy devices and protocols is demanded [17,19] to ensure the best method for collecting activity data in depressed subjects. In addition, utilisation of only one single sensor at the wrist limits qualitative analyses of motor activity. For example, motor activity measured at the wrist can result from gestures or from arm swings while walking [20]. Thus, more information is necessary to understand the abnormalities in motor activity in patients with depression.

Second, Burton et al. [17], among others [21–23], call for an improvement of analytic methods in order to ensure that all relevant features are utilised when categorising behaviour types. For instance, movement patterns like sitting, standing and lying are features which can be extracted from an acceleration signal quite easily. Yet, most of the studies reported solely captured or calculated global motor activity counts from acceleration data, thereby not taking a deeper look at the actual behaviour. Further, motor activity is generally characterised by amplitude and frequency. Thus, clarifying if patients walk less (amplitude) or slower (frequency) than healthy controls requires up to date analytic methods.

Third, Burton et al. [17] request standardised methods to detect diurnal variation, thereby figuring out the most suitable timeframe to distinguish abnormalities in motor activity of patients suffering from MDD compared to healthy controls, for example. Similarly, no recommendations are available to date.

To overcome the methodological limitations voiced by Burton et al. [17], we monitored motor activity using the non-dominant wrist as in most previous studies [17] and additionally at the centre of mass (chest) for 24 hours. Subjects were in-patients suffering from MDD, which were assessed at the beginning (MD) and after four weeks of inpatient treatment (MD-post), as well as healthy controls (HC). We used sophisticated methods to analyse the activity data and did take diurnal variation into account.

In order to replicate findings from Burton et al.’s [17] systematic review, we hypothesised lower motor activity measured at the wrist in MD compared to HC (hypothesis I). To clarify the best sensor placement, analytic strategy and diurnal timeframe to detect differences between MD and HC, we performed multiple explorative analysis differentiating MD and HC regarding: a) sensor placements (wrist- vs. chest-accelerometry) and extracted features (pure acceleration vs. amplitude and frequency extracted using spectral analysis) as well as b) diurnal variation of motor activity, motion patterns (sitting/standing vs. lying and walking) and posture (chest inclination).

Again, to replicate findings from Burton et al.’s [17] systematic review, we hypothesised increased motor activity assessed at the wrist in MD-post compared to MD-pre (hypothesis II). To clarify the most adequate sensor placement, analytic strategy and diurnal timeframe to detect differences between MD-pre and MD-post, we performed multiple explorative analysis investigating: a) sensor placements and extracted features as well as b) diurnal variation of motor activity, motion patterns and posture.

## Methods

### Design

The study was conceptualised both as a case-control and pre-post treatment design. Motor activity of patients suffering from MDD was assessed at the beginning (MD) and after 4 weeks of hospitalisation (MD-post); a comparison group of healthy controls (HC) was assessed once.

### Study participants

The sample consisted of 27 in-patients (MD: 12 female, 15 male) suffering from MDD and a comparison group of 16 healthy adults (HC: 6 female, 10 male). The difference in the sex ratio and the mean age of the two groups were not significantly different with 39.6 years for MD (range = 19–64, SD = 12.4) and 41.3 years for HC (range = 21–62, SD = 12.3). Moreover, there was no significant difference in the BMI [kg/m^2^] of MD (M = 25.7, range = 16.5–38.2, SD = 5.5) and HC (M = 27.2, range = 19.5–36.4, SD = 5.3).

Motor activity data from all patients were available at the beginning of in-patient treatment, whereas after 4-weeks of in-patient treatment only 18 out of the 27 patients were measured. Drop-out analyses revealed no significant differences between patients remaining in the study and drop-outs regarding age, gender, BMI, HDRS-Score and mean motor activity. In order to compare motor activity of untreated patients with HC, MD (n = 27) vs. HC (n = 16) were taken into account. In order to analyse treatment effects only patients participating at both assessments were included (n = 18).

#### Patients

Patients were recruited within the study SOLID (“Stress, Obesity, Liverfat, Insulin Resistance in Depression”, German Clinical Trials Register—ID: DRKS00004324, http://drks-neu.uniklinik-freiburg.de/drks_web/navigate.do?navigationId=trial.HTML&TRIAL_ID=DRKS00004324) at the Clinic for Psychiatry and Psychotherapy of the Central Institute of Mental Health (ZI) in Mannheim.

Patients attending the clinic with unipolar disorder or bipolar disorder were included in the study if they were in a moderate to severely depressive state and between 18 and 65 years of age. However, even though bipolar disorder was not an exclusion criterion in the SOLID study, we only assessed motor activity in patients with unipolar depression. Diagnoses were given following clinical psychiatric examination and chart review. Severity of depression was assessed by the treating psychiatrists according to the 21-item Hamilton Depression Rating Scale (HDRS)—at least 18 points right after admission were mandatory to be included in the study. Psychiatrists carried out all the HDRS-ratings at pre-assessment (MD) and at post assessment (MD-post). There was a significant difference [t(17) = 11.48, p<0.001] in the HDRS-Scores between MD-pre (M = 23.6, SD = 4.6) and MD-post (M = 11.4, SD = 5.9).

Exclusion criteria were a current psychotic episode, a depressive episode of organic nature, dementia, current use of steroids, alcohol or sedative dependency, or surgery in the last six months. Patients with a significant risk of suicidal behaviour were excluded, especially when they had active suicidal ideations with some intent to act. Patients with non-specific active suicidal thoughts were not excluded. In addition, patients of consent or legal incapacity and patients suffering from anaemia were also excluded.

Patients obtaining psychotropic drugs prior to their hospitalisation received a five-day wash out period without pharmacological treatment carried out prior to our study [24]. At the time of second measurement, they received various types, combinations and doses of psychopharmacological treatment.

#### Controls

A control group of healthy individuals comparable to the patient group regarding gender, age and body-mass-index was recruited via a telephone list of interested study participants. Participants with a current mental disorder, prior psychiatric disorders or illness influencing motor activity were excluded. Furthermore, participants pursuing a job with high motor activity demands (e.g. construction worker) were also excluded. Thus, the control group comprised participants performing an office job (e.g. chemist, architect, research assistant), studying (e.g. pupil, university student) or staying at home (e.g. house-wife, pensioner) at the time of the assessment. They did not work shifts or suffer from sleep deprivation. To determine mental disorders, the control group underwent the Mini International Neuropsychiatric Interview (M.I.N.I., Version 5.0.0) for DSM-IV and ICD-10 [25].

### Assessment of motor activity

Each participant carried a portable physiological recorder-analyser system, the Varioport-B (Becker Engineering, Karlsruhe, Germany). The device with a size of 120x65x22mm and a weight of 170g enables the attachment of different sensors (e.g. Accelerometers, ECG, EMG, EDA, etc.) with a maximum of 34 recording channels. The data is stored on a 4GB SD-Card. Standard rechargeable batteries enable a maximum operation period of 100 hours (for details see [26,27]). Motor activity was sampled at 32 Hz over a minimum period of 24 hours by 2 accelerative sensors placed on the chest (3-dimensional accelerometry) and on the non-dominant wrist (1-dimensional accelerometry). Patients’ motor activity was assessed for 24 hours at the beginning of inpatient treatment and—in patients obtaining psychotropic drugs prior to their hospitalisation—after a 5-day wash-out period. In addition, motor activity of patients was measured in infirmary for 24 hours after four weeks of pharmacological anti-depressive treatment. The HC group wore the device for 24 hours in everyday life.

After the application of the portable physiological recorder system by a supervisor, each participant was asked to adopt several motion patterns (seated, standing, lying on the back, lying on the left side, lying on the right side and walking) for approximately 35 seconds according to the standard protocol of the Freiburger Monitoring System [28]. The participants were instructed to carry on with their usual activities (habitual behaviour) during the observation period. In addition, they were asked to note particular physical activities and sport activities in a protocol.

### Data analysis

All data analyses were computed separately for both accelerometers attached to the wrist and the chest and for each sensor axis (one axis of the wrist accelerometer and three axes of the chest accelerometer: vertical, horizontal and sagittal).

First, motor activity was separated offline into AC and DC components by a FIR digital filter with a cut-off frequency at 0.5 Hz. Raw signal, DC values, and rectified AC values were averaged across data points for each condition and monitoring segment. In addition, the vector (cumulative acceleration) of the three axes of the chest sensor was computed following the rules of vector addition. All online and offline analyses and artefact checks were performed by the interactive software package “Freiburg Monitoring System” [28] according to a published procedure [29].

**Fig 1:**
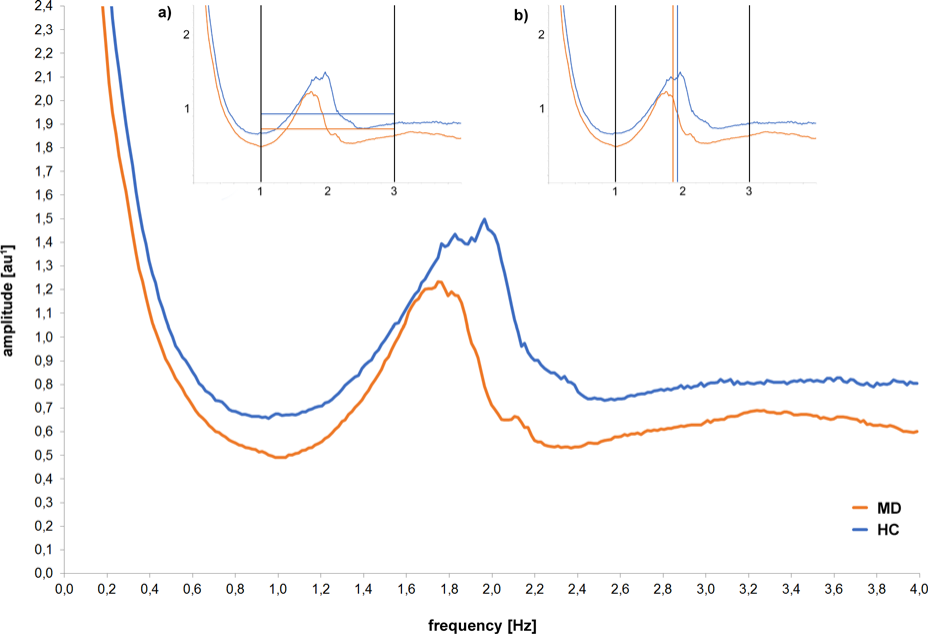
Spectral analysis of acceleration (frequency and amplitude) of MD patients at the beginning of treatment (MD) and healthy controls (HC) captured with the vertical axes of the chest-accelerometer. Mean amplitude values (in au^1^; grey and black horizontal resp.) as well as the group centroid frequency (in Hz; grey and black vertical line resp.) for the whole spectrum of walking (1–3Hz) are depicted in Fig 1a, 1b, respectively. ^1^arbitrary unit: values are based on g but attenuated due to the computation of the spectrum and the nonstationarity of the data.

**Fig 2:**
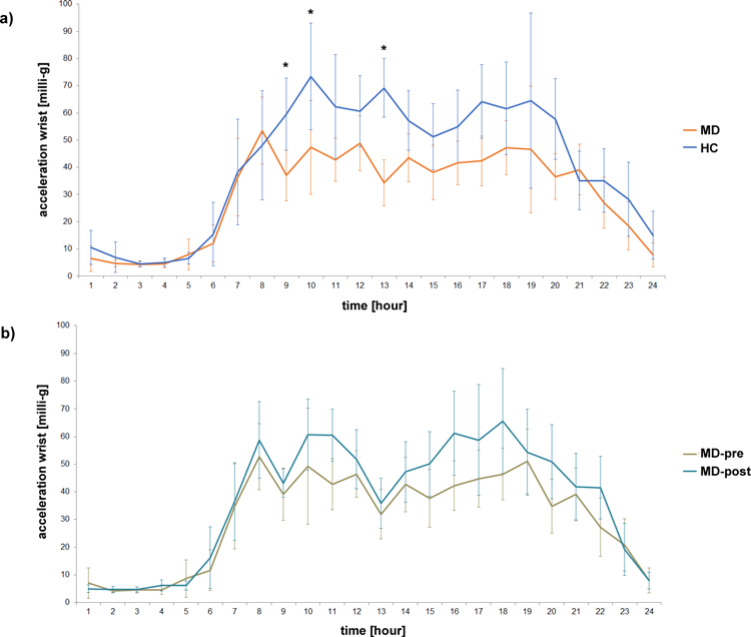
Diurnal variation of motor activity measured at the wrist a) in MD patients at the beginning of treatment (MD) and healthy controls (HC) as well as b) in MD patients at the beginning (MD-pre) and after 4 weeks of inpatient treatment (MD-post). * show significant differences in hours analysed with post-hoc test (Newman-Keuls).

**Fig 3:**
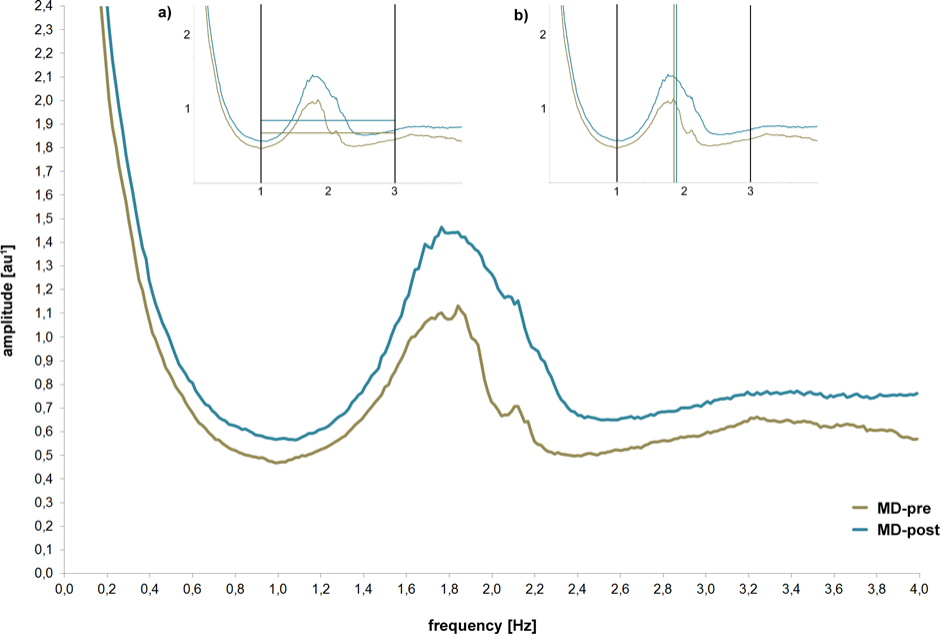
Spectral analysis of acceleration (frequency and amplitude) of MD patients at the beginning (MD-pre) and after 4 weeks (MD-post) of inpatient treatment captured with the vertical axes of the chest-accelerometer. Mean amplitude values (milli-g; grey and black horizontal lines resp.) as well as the group centroid frequency (in Hz; grey and black vertical lines resp.) for the whole spectrum of walking (1–3Hz) are depicted in Fig 3a, 3b, respectively. ^1^arbitrary unit: values are based on g but attenuated due to the computation of the spectrum and the nonstationarity of the data.

Second, mean daily motor activity (acceleration values measured in milli-g) over the 24 h period as well as the hourly means of motor activity were calculated using SPSS version 21. Third, raw activity data was segmented into 60-s periods and subjected to Fourier-based spectral analysis using Vision Analyser version 1.04 software (Brain Products, Gilching, Germany, http://www.brainproducts.com). For further statistical analysis, the mean amplitude (see Figs 1a and 2a) and the centroid frequency (dividing the area of the spectrum into two equal parts; see Figs 1b and 2b) were computed within the frequency bands from 1 to 3 Hz. The frequency range 1–3 Hz was used as it covers the main motor activity within our groups (see Figs 1 and 3) and enabled us to focus gross motor activity, like walking and running. The amplitude of the spectral analysis is an arbitrary unit (au): the values are based on g but attenuated due to the computation of the spectrum and the nonstationarity of the data.

Fourth, the standard protocol data was used to calculate individual milli-g-cut-off-values and thereby to identify the duration of participant’s motion patterns (in hours) divided into the situations sitting/standing, lying and walking within the 24 h periods; these analyses were conducted following the principle of a published procedure [28]. Fifth, mean daily motor activity (AC values measured in milli-g) over the 24 h were computed for the situations sitting/standing, lying and walking.

### Statistical analysis

To investigate group differences, t-tests and effect sizes (Cohen’s d) were used after data were tested for normality. Logistic regression analyses were used to predict the affiliation of participants to the group of subjects (MD vs. HC) and thereby to identify the best feature-placement combination. We used the forward method including only the most influential predictors. The reported measure Nagelkerges R² adjusts Cox and Snell R² to enable a maximum value of 1. Cox and Snell R² is based on log likelihoods taking sample size into account. Thus, Nagelkerges R² is the most analogous measure to R² in linear Regression, provided in SPSS [30]. Two-factorial ANOVAs were used to calculate group (MD vs. HC; MD-pre vs. MD-post) and time (24 hours) effects in diurnal variation; post-hoc analyses were conducted using the Newman-Keuls test. SPSS version 21 was used for all analysis; p-values below 0.05 were considered significant.

### Ethical consideration

The study was approved by the medical ethics committee II of the Medical Faculty Mannheim at the Ruprecht-Karls-University in Heidelberg. It fulfilled the ethical guidelines for medical research according to the declaration of Helsinki. Written and oral information about study procedures were presented to all eligible participants before written informed consent was obtained. Experienced psychiatrists excluded patients with insufficient capacity to provide informed consent from the study. There was no surrogate consent procedure. All participants were free to withdraw from the study at any time. The participants of the HC group were paid for participating in the study.

## Results

### Motor activity in patients at the beginning of treatment (MD) versus Healthy Controls (HC)

Analyses revealed a significant difference of motor activity assessed at the wrist (see Table 1: acceleration wrist) between MD and HC (p = 0.001), in which MD (30.35 milli-g) showed 26% lower motor activity than HC (41.22 milli-g). Having received comparable findings to previous studies [17], we were able to apply meaningful analyses on details of motor activity, like sensor placement, features and diurnal variation.

**Table 1:** Differences in mean motor activity and motion patterns over a 24 hour period among MD patients in the beginning of treatment (MD) and healthy controls (HC)—itemised in several feature-placement combinations measured in different accelerometer axes and sorted according to effect sizes by Cohen.

feature-placement combinations	MD (SD)	HC (SD)	t	df	p	d
acceleration wrist [milli-g]	30.35 (7.74)	41.22 (7.92)	-4.42	41	0.001***	1.39
amplitude^1^ wrist [au^2^]	1.64 (0.48)	2.18 (0.41)	-3.78	41	0.001***	1.22
amplitude^1^ chest vertical [au^2^]	0.72 (0.18)	0.93 (0.19)	-3.72	41	0.001***	1.16
amplitude^1^ chest horizontal [au^2^]	0.61 (0.13)	0.79 (0.18)	-3.69	41	0.001***	1.12
acceleration chest (vector)^3^ [milli-g]	49.87 (10.20)	61.19 (10.92)	-3.43	41	0.001***	1.07
centroid frequency^1^ chest vertical [Hz]	1.85 (0.07)	1.92 (0.07)	-3.31	41	0.002**	1.04
acceleration chest horizontal [milli-g]	17.31 (3.42)	20.95 (3.64)	-3.64	41	0.002**	1.03
acceleration chest sagittal [milli-g]	19.53 (3.46)	23.03 (3.63)	-3.15	41	0.003**	0.99
acceleration chest vertical [milli-g]	16.77 (4.33)	20.91 (4.69)	-2.94	41	0.005**	0.92
amplitude^1^ chest sagittal [au^2^]	0.66 (0.13)	0.78 (0.14)	-2.84	41	0.007**	0.89
centroid frequency^1^ chest sagittal [Hz]	1.77 (0.05)	1.81 (0.05)	-2.51	41	0.016*	0.79
time spent reclined [hours]	10.09 (1.99)	8.62 (2.11)	2.29	41	0.027*	0.72
time spent walking [hours]	3.53 (1.36)	4.37 (1.26)	-2.02	41	0.050*	0.64
centroid frequency^1^ wrist [Hz]	1.82 (0.03)	1.83 (0.03)	-1.41	41	0.166, n.s.	0.45
centroid frequency^1^ chest horizontal [Hz]	1.99 (0.06)	1.97 (0.05)	1.18	41	0.246, n.s.	0.38
time spent sitting/standing [hours]	10.39 (1.79)	11.01 (2.26)	-0.99	41	0.327, n.s.	0.30
acceleration wrist during walking [milli-g]	98.66 (14.74)	120.58 (13.95)	-4.81	41	0.000, n.s.	1.53
acceleration wrist during sitting/standing & lying [milli-g]	18.62 (5.24)	23.54 (4.91)	-3.05	41	0.004, n.s.	0.97

Abbreviations: SD: standard deviation; n.s. = not significant;

*p≤0.05,

**p≤0.01,

***p≤0.001;

d: Cohen’s effect size.

^1^calculated with spectral analysis;

^2^arbitrary unit: values are based on g but attenuated due to the computation of the spectrum and the nonstationarity of the data;

^3^cumulative acceleration of the three axes of the chest sensor computed following the rules of vector addition.

### Sensor placement and extracted features

To identify the most powerful parameter for distinguishing motor activity between MD and HC, we extracted different features, like mean acceleration, amplitude and centroid frequency from spectral analysis, and motion patterns across different placements (wrist vs. chest). As stated above, mean acceleration is the mean intensity of the acceleration signal over time, representing mean motor activity. Amplitude and centroid frequency [Hz] are features extracted using spectral analysis.

As shown in Fig 1, spectral analyses deconstruct the signal into amplitude (y-axis) and frequency (x-axis). Descriptively, Fig 1 depicts the vertical sensor axis (chest) showing higher amplitudes (y-axis) in HC (grey line) across the entire frequency spectrum (x-axis). As represented in Fig 1a, mean amplitude was heightened in HC compared to MD (grey and black horizontal lines respectively). Analysis was limited to the range of 1 to 3 Hz, which represented the spectrum of slow walking to running. Fig 1b represents higher frequencies (x-axis) in HC compared to MD, illustrated in the curve shifted to the right. This can be quantified by calculating the centroid frequency [Hz], which divides the frequency spectrum into two equal parts. Descriptively, the centroid frequency (grey vs. black vertical lines) was heightened in HC compared to MD. In practice, this means that HC showed higher amplitudes (Fig 1a) as well as higher frequencies (Fig 1b) than MD—suggesting they might walk more and faster.

Table 1 shows the statistics of comparing groups regarding multiple extracted features across placements (feature-placement combinations). Feature-placement combinations are sorted by their power to differentiate groups (effect size). Out of the 16 calculated feature-placement combinations, 13 revealed a significant difference between MD and HC, showing higher motor activity in HC (even with Bonferroni adjustment, 12 achieved a significant p value). The acceleration of the wrist did show the highest effect size in distinguishing both groups (d = 1.39). In addition, the mean amplitude of the wrist (d = 1.22) as well as that of the chest sensor (vertical axis) showed high effect sizes (d = 1.16). The vertical axis of the chest sensor showed the highest effect size regarding frequency differences (d = 1.04).

The inference statistics confirmed our descriptive findings from Fig 1a and 1b, revealing significant heightened amplitude [t(41) = -3.72, p = 0.001] and centroid frequency [t(41) = -2.94, p = <0.005] in HC compared to MD (see Table 1: amplitude chest vertical and centroid frequency vertical). In numbers, motor activity of HC compared to MD was quantitatively (amplitude) enhanced by 25% (1.64 milli-g/2.18 milli-g; d = 1.16). Furthermore, we detected qualitative (centroid frequency) heightened motor activity in HC (d = 1.04). Looking at the motion patterns, analysis revealed that HC spent on average 1.47 (8.62 vs. 10.09) hours less time per day lying than MD (p<0.05, d = 0.72). Furthermore, HC walked on average 0.84 (4.37 vs. 3.53) hours more per day than MD (p = 0.05, d = 0.64).

In addition, we used multiple logistic regressions to investigate whether and which feature-placement combination did show an additional contribution when differentiating between groups (see Table 2). In a first step, we added all acceleration-features to our most prominent feature “acceleration wrist”, but none of the additional acceleration-features were significant (Nagelkerkes R^2^ = 0.42). In a second step, we added all amplitude-features in the range of 1–3 Hz to our most prominent feature “acceleration wrist”, but none of the additional amplitude-features were significant (Nagelkerkes R^2^ = 0.42). In a third step, we added all centroid-features calculated in the range of 1–3Hz to our most prominent feature “acceleration wrist”; here only the centroid frequency of the chest (vertical axis) revealed significance and improved the model (descriptively best model Nagelkerkes R^2^ = 0.54 with the highest prediction accuracy of 79.1%).

**Table 2:** Logistic regression analysis to predict the affiliation of participants to the group of subjects (MD patients at the beginning of treatment (MD) vs. healthy controls (HC)) combining several calculated feature-placement combinations; significant combinations (*) are highlighted in bold characters.

feature-placement combinations	Nagelkerkes R^2^	prediction accuracy [%]
acceleration wrist [milli-g]* + acceleration chest (vector)^1^ [milli-g] + acceleration chest vertical [milli-g] + acceleration chest sagittal [milli-g] + acceleration chest horizontal [milli-g]	0.424	76.7
acceleration wrist [milli-g]* + amplitude^2^ wrist [au^3^] + amplitude^2^ chest vertical [au^3^] + amplitude^2^ chest sagittal [au^3^] + amplitude^2^ chest horizontal [au^3^]	0.424	76.7
acceleration wrist [milli-g]* + centroid frequency^2^ wrist [Hz] + ***centroid frequency*** ^*2*^ chest vertical [Hz]* + centroid frequency^2^ chest sagittal [Hz] + centroid frequency^2^ chest horizontal [Hz]	0.540	79.1

^1^cumulative acceleration of the three axes of the chest sensor computed following the rules of vector addition;

^2^calculated with spectral analysis;

^3^arbitrary unit: values are based on g but attenuated due to the computation of the spectrum and the nonstationarity of the data.

#### Diurnal variation, motion patterns and posture

Fig 2a shows the diurnal variation of acceleration (wrist) in MD and HC. Descriptively, motor activity was lower in MD during daytime, but comparable to HC at night. A two-factorial ANOVA showed a significant interaction [F(23,94) = 2.64, p<0.001] between time and group. We also found significant group [F(1,41) = 19.1, p<0.001] and time [F(23,94) = 39.36, p<0.001] effects. Post-hoc-tests (Newman-Keuls) showed significant differences in the hours 9, 10 and 11 (as shown in Fig 2a).

To examine whether differences in activity between MD and HC measured at the wrist resulted from gesture or from arm swing during walking, we calculated the mean acceleration values measured at the wrist during walking as well as those measured during sitting/standing and lying. The data captured during walking revealed an effect size about one third higher compared to the acceleration values measured while HC and MD were not walking (d = 1.53 vs. 0.97; see Table 1).

In addition, in explorative analysis HC showed a more upright posture compared to MD during sitting and standing (assessed with the vertical axis of the chest sensor). Although Student’s t-Test showed no significant difference [t(41) = 1.52, p = 0.136], we found a medium effect size (d = 0.50) suggesting that MD leaned forward more in comparison to HC (9 degrees difference in the vertical body axis) within the motion patterns sitting and standing.

### Motor activity in MDD: pre (MD-pre) versus post (MD-post) treatment

Motor activity assessed at the wrist differed significantly (p>0.05) among MD-pre (30.49 milli-g) and MD-post (37.04 milli-g), with 18% higher motor activity in MD-post (see Table 3: acceleration wrist). This result is in line with previous research [17]. Additionally, standard deviation of motor activity in MD-post was increased (see Table 3: acceleration wrist). Indeed, only 12 out of 18 MD-pre patients showed heightened motor activity (acceleration wrist) after the treatment (MD-post), reflecting 67% of the sample.

**Table 3:** Differences in mean motor activity and motion patterns over a 24 hour period among MD patients in the beginning (MD-pre) and after 4 weeks (MD-post) of in-patient treatment—itemised in several feature-placement combinations measured in different accelerometer axes and sorted according to effect sizes by Cohen.

feature-placement combinations	MD-pre (SD)	MD-post (SD)	t	df	p	d
acceleration chest horizontal [milli-g]	16.93 (3.54)	20.78 (5.68)	-2.68	17	0.02*	0.63
amplitude^1^ chest horizontal [au^2^]	0.60 (0.15)	0.74 (0.22)	-2.60	17	0.02*	0.61
centroid frequency^1^ wrist [Hz]	1.83 (0.03)	1.85 (0.03)	-2.58	17	0.02*	0.61
acceleration wrist [milli-g]	30.49 (8.82)	37.04 (10.11)	-2.33	17	0.03*	0.55
acceleration chest vertical [milli-g]	16.33 (4.09)	20.75 (7.42)	-2.32	17	0.03*	0.55
acceleration chest (vector)^3^ [milli-g]	48.71 (11.03)	58.13 (15.93)	-2.30	17	0.03*	0.54
acceleration chest sagittal [milli-g]	19.16 (3.79)	22.38 (5.51)	-2.28	17	0.04*	0.54
amplitude^1^ chest vertical [au^2^]	0.69 (0.18)	0.86 (0.32)	-2.25	17	0.04*	0.53
amplitude^1^ chest sagittal [au^2^]	0.64 (0.15)	0.75 (0.20)	-2.12	17	0.05*	0.50
amplitude^1^ wrist [au^2^]	1.67 (0.54)	1.99 (0.61)	-2.07	17	0.05*	0.49
centroid frequency^1^ chest sagittal [Hz]	1.79 (0.05)	1.81 (0.06)	-2.02	17	0.06, n.s.	0.48
time spent reclined [hours]	10.34 (2.19)	9.44 (1.25)	1.79	17	0.09, n.s.	0.42
centroid frequency^1^ chest vertical [Hz]	1.86 (0.08)	1.90 (0.10)	-1.68	17	0.11, n.s.	0.40
time spent walking [hours]	3.55 (1.40)	3.91 (1.42)	-1.23	17	0.24, n.s.	0.29
time spent sitting/standing [hours]	10.11 (1.55)	10.65 (1.60)	-1.20	17	0.25, n.s.	0.28
centroid frequency^1^ chest horizontal [Hz]	1.98 (0.06)	1.98 (0.06)	-0.12	17	0.91, n.s.	0.03

Abbreviations: SD: standard deviation; n.s. = not significant;

*p≤0.05;

d: Cohen’s effect size.

^1^calculated with spectral analysis;

^2^arbitrary unit: values are based on g but attenuated due to the computation of the spectrum and the nonstationarity of the data;

^3^cumulative acceleration of the three axes of the chest sensor computed following the rules of vector addition.

#### Sensor placement and extracted features

As shown in Fig 3, MD-post reached higher amplitudes (y-axis) across the whole frequency spectrum (x-axis) compared to MD-pre. In the range of 1 to 3 Hz, statistics confirmed our descriptive finding, showing significant differences [t(17) = -2.25, p<0.05] with 21% (16.33 milli-g/20.75 milli-g) elevated motor activity in MD-post (see Table 3: amplitude chest vertical). However, differences in the centroid frequency (vertical sensor chest) lacked statistical significance (p = 0.11) and revealed only a small effect size (d = 0.40).

In Table 3, statistics distinguishing motor activity of MD-pre and MD-post by using different feature-placement combinations are provided. Feature-placement combinations are sorted by their power to differentiate MD-pre vs. MD-post (effect size). 10 out of 16 calculated feature-placement combinations (including the mean acceleration measured at the wrist) revealed significant differences, showing higher motor activity in MD-post compared to MD-pre. In contrast to the comparison of motor activity between MD and HC (see Table 1), we determined only mean effect sizes illustrating the magnitude of differences in motor activity of MD-pre compared to MD-post (see Table 3: e.g. acceleration chest horizontal (d = 0.63)).

#### Diurnal variation, motion patterns and posture

Fig 2b shows differences in the diurnal variation of motor activity captured at the wrist comparing MD-pre and MD-post. Descriptively we found higher activity in MD-post compared to MD-pre throughout daytime, but no difference during night time. However, analysis of variance revealed no overall interaction effect [F(23,39) = 1.20, p = 0.242], but significant effects for MD-pre vs. MD-post [F(1,17) = 5.37, p<0.05] and time [F(23,39) = 36.46, p<0.001].

MD-post spent on average 0.9 (9.44 vs. 10.34; d = 0.42) hours less time per day in a reclined position than MD-pre. Moreover, MD-post walked on average 0.4 (3.91 vs. 3.55; d = 0.29) hours more per day than MD-pre. These findings lacked significance (see Table 3). Nevertheless, they are in line with our findings comparing MD and HC (see Table 1).

Furthermore, MD-post revealed a more upright posture compared to MD-pre during sitting and standing, with MD-pre being leaned forward in comparison to MD-post (4 degrees difference in the vertical body axis). However, this difference is statistically not significant [t(17) = 1.23, p = 0.235] and effect size is small (d = 0.29).

## Discussion

As hypothesised, MD showed lower motor activity measured at the wrist compared to HC (hypothesis I). This result is in line with earlier studies and systematic reviews [3–6,17], enabling us to perform multiple analyses in order to clarify which sensor placement, analytic strategy and diurnal time frame best differentiated MD from HC. Looking at single feature-placement combinations, our data showed that almost all of the calculated feature-placement combinations showed significant differences between MD and HC. This implies that altered motor activity in depressed patients is not limited to alterations in single extremities, like arm movements, but comprises whole body activity. Mean acceleration measured at the wrist was the most powerful parameter for distinguishing groups, evidenced by the highest effect size. This is advantageous, as most previous studies used the wrist for sensor placement [17]. On a theoretical level, the suitability of wrist placement can be explained by capturing both movement of the whole body (like walking vs. sleeping) and motor activity of the arms (like arm swings or gestures). In comparison, a device attached to the centre of mass would only be able to capture movement of the chest or hip. Overall, this is in line with several of our findings, showing higher effect sizes when the respective parameter contained both core body movement and activity of the arms, like arm swing during sitting (pure gesture) vs. arm swing during walking. Summing up, motor retardation in MD-patients was not only reflected by gross motor activity, but also in broad domains like gait, gestures and probably fine motor behaviour.

Taking a closer look at the analytic strategies, we used spectral analyses to investigate movement frequencies, providing evidence that both amplitude and frequency were altered in MD compared to HC. In practice, MD walked less and slower compared to HC. This is in line with earlier studies showing altered gait patterns, like reduced gait velocity, in depressed subjects compared to HC [20,31,32]. This finding was confirmed by our analyses on motion patterns showing that MD spent less time walking and more time lying than HC. Applying logistic regressions, we could also show that the combination of a frequency parameter with wrist acceleration differentiated groups best. Therefore, in addition to the mean acceleration of gross motor activity, which is an expression for the amount of activity, the frequency/speed seems to be an additional important manifestation of MDD.

Looking at the diurnal variation, differences of motor activity between groups could be described during daytime but not night time. In addition, data descriptively did not show disturbances in circadian rhythm, as reported in several studies [33], like postponed sleeping phase [34]. However, our depressed sample consisted of in-patients participating in daily clinical routines, thereby not being able to choose their own stand-up time, for example.

Regarding postures, we investigated whether MD and MD-pre were more inclined forward while sitting and standing throughout the 24 hour period compared to HC and MD-post, respectively, which would be in line with embodiment theories [35] and studies showing relations between posture and cognitive biases in depression [36]. We found differences in numbers that did however lack significance. According to the small sample size, we did not differentiate between subsamples regarding retardation or agitation or regarding treatment success, which might help distinguish group differences. In addition, fixing the sensor to the head, compared to the chest, might be better at capturing inclined posture.

Results comparing MD patients at the beginning (MD-pre) vs. after treatment (MD-post) fit our findings comparing MD vs. HC. Motor activity measured at the wrist after 4 weeks of treatment was significantly enhanced (hypothesis II), a finding in line with previous research [5,7–10,17].

In order to clarify the most adequate sensor placement, analytic strategy and diurnal time frame to distinguish between patients at the beginning and end of treatment we performed multiple analyses. Similar to the comparison between MD and HC, most of the calculated feature-placement combinations revealed significant differences between MD-pre and MD-post. We detected smaller effect sizes in most feature-placement combinations, compared to MD vs. HC. This was expected as not all of the patients showed enhanced motor activity at the end of treatment.

Using spectral analysis to analyse movement frequencies showed that only amplitude but not frequency was enhanced after 4 weeks of in-patient treatment. The finding of enhanced amplitude of motor activity was supported by heightened walking time next to reduced time spent in a reclined position. In sum, motor activity in MD-post compared to MD-pre was only increased with regard to amplitude but not to frequency, implying that patients walked more but not faster after 4 weeks of in-patient treatment. Whether this was caused by a general lower walking speed in people prone to depression or by the fact that not all patients in our sample showed enhanced motor activity at the end of treatment, cannot be determined by our study and warrants further investigation. Looking at the diurnal variation, differences of motor activity between groups appeared during daytime but not during night time. However, we did not find significant differences for specific hours during the day.

In total, our findings lead to two main conclusions: First, using additional sensor-placements and improving analytic strategies is worthwhile for research aiming to examine details of altered motor activity in depressed subjects. For instance, to differentiate motion patterns and thereby to discriminate between motor activity of the whole body and motor activity of the arms, it is crucial to attach sensors to the arm and the trunk. To distinguish amplitude vs. frequency, which can be translated to walking quantity vs. speed, it is necessary to use appropriate analytic strategies like spectral analysis. Second, we could show that attaching the sensor to the wrist, as done in most existing studies [17], is most powerful when differentiating between MD and HC. Measuring acceleration at the wrist seems to be sufficient to indicate general abnormalities in motor activity of depressed patients as required in clinical practice, studies examining treatment success or feedback interventions [37,38] for example.

Some limitations of our study should be mentioned. First, even though our sample size was relatively small, we could show significant differences, as hypothesised. Group sizes (MD vs. HC) varied and groups were not matched for age and sex. However, statistical analyses revealed no significant differences between groups regarding age and sex. In spite of the fact that equally large and matched groups would have been preferable, medium to high effect sizes enabled us to perform detailed methodological analyses depicting the best sensor placement, analytic strategy and diurnal time frame. Second, we used clinical psychiatric examination and chart review to confirm the existence of a depressive episode, which hampers the report of inter-rater reliability. Furthermore, the HDRS was assessed by the treating psychiatrists and not by independent raters. However, diagnoses were made by experienced psychiatrists and an additional inclusion criterion was the severity of depression (>18 points on HDRS). Third, patients were in-patients taking part in daily clinical routine, whereas HC were living at home. Even though empirical evidence is sparse, clinical routine of patients might led to artificially affected motor activity, limiting between group differences. However, we found significant differences with high effect sizes distinguishing both groups and the main focus of the paper was to clarify methodological issues (sensor placement, analytic strategy, diurnal time). Fourth, motivated by our methodological focus we used a highly demanding study protocol. Patients wore multiple sensors, connected by wires, and had to perform a standard protocol at the beginning of the assessment. To limit participant burden, we only monitored two episodes 24 hours in length. For future studies, which do not focus on methodological issues, we would recommend assessing longer time frames, including working days and weekend days [19]. Fifth, as the main focus of our study dealt with methodological issues, we did only include patients suffering from MDD and HC. However, abnormalities in motor activity are seen in other psychiatric disorders, too (e.g. reduced motor activity in residual schizophrenia or enhanced motor activity within manic episodes of patients suffering from bipolar disorder [1,2]). Therefore, future studies should include other patient groups to clarify specificity.

In conclusion, our study showed decreased motor activity in patients suffering from MDD compared to HC and increased motor activity after treatment compared to the beginning. Furthermore, our findings showed that measuring acceleration at the wrist was sufficient to indicate abnormalities in motor activity of depressed patients. However, application of additional sensors and more sophisticated analytic strategies are worthwhile to detect more specific differences of altered motor activity.
